# PD32 Opportunities And Challenges: Health Technology Assessment Body Perspectives On The Integration Of Artificial Intelligence In Evidence Synthesis

**DOI:** 10.1017/S0266462325101669

**Published:** 2025-12-29

**Authors:** Grace Fox, Juliette Torres Ames, Nita Santpurkar, Petra Nass, Emanuele Arcà

## Abstract

**Introduction:**

Evidence synthesis is the foundation of health technology assessment (HTA); however, systematic literature reviews (SLRs) are highly resource intensive. While augmentation with artificial intelligence (AI) theoretically offers to make this rigorous process more efficient, it is unclear whether AI-supported SLRs will be widely accepted among HTA bodies. This study’s objective was to evaluate how HTA bodies regard the use of AI-supported SLRs.

**Methods:**

A targeted literature review (TLR) (January 2019 to October 2024) was conducted in Embase, MEDLINE, and the gray literature. Search terms included AI, natural language processing, large language models, and machine learning. The TLR informed development of a survey to be fielded to respondents from HTA bodies and questions for qualitative interviews.

**Results:**

The TLR found that most HTA bodies do not address using AI for SLRs. Two that do are the National Institute for Health and Care Excellence (England) and the Institute for Quality and Efficiency in Health Care (Germany). Both suggested that AI can support human efforts across multiple SLR phases. Respondents from Europe and the USA completed the survey and qualitative interviews. Most respondents had some familiarity with using AI in SLRs but said their respective organizations doubted AI’s utility in improving the quality of SLRs. Respondents stated the primary responsibility for AI tool development and validation should not rest with manufacturers.

**Conclusions:**

Most HTA bodies do not address using AI for SLRs, but respondents said that AI might improve SLR production by augmenting (not replacing) human effort. Gaining acceptance for AI use in SLRs for HTA will require multistakeholder engagement to ensure transparency and reliability. HTA bodies will need appropriate infrastructure and legal frameworks so that they can test and use AI software.

